# Targeting Ceramides and Adiponectin Receptors in the Islet of Langerhans for Treating Diabetes

**DOI:** 10.3390/molecules27186117

**Published:** 2022-09-19

**Authors:** Wen-hong Li

**Affiliations:** Departments of Cell Biology and of Biochemistry, University of Texas Southwestern Medical Center, Dallas, TX 75390-9039, USA; wen-hong.li@utsouthwestern.edu; Tel.: +1-214-648-3879

**Keywords:** ceramides, sphingolipid, ceramide synthase, adiponectin, adiponectin receptor, pancreatic islets, beta cell, lipotoxicity, beta cell apoptosis, AdipoRon, AdipoRonPEG5, ceramidase, zinc, diabetes

## Abstract

Ceramides belong to the sphingolipid family and represent the central hub of the sphingolipid network. In obesity, oversupply of saturated fatty acids including palmitate raises ceramide levels which can be detrimental to cells. Elevated ceramides can cause insulin resistance, endoplasmic reticulum stress, and mitochondrial dysfunction. Studies over the last few decades have highlighted the role played by ceramides in pancreatic islet β-cell apoptosis, especially under glucolipotoxic and inflammatory conditions. This review focuses on ceramides and adiponectin receptor signaling, summarizing recent advancements in our understanding of their roles in islet β-cells and the discovery of zinc-dependent lipid hydrolase (ceramidase) activity of adiponectin receptors. The therapeutic potential of targeting these events to prevent islet β-cell loss for treating diabetes is discussed.

## 1. Introduction

Diabetes mellitus (DM) is increasing at an alarming rate and is approaching pandemic level worldwide. The Centers for Disease Control and Prevention (CDC) estimated that >34 million people in the US (10.5% of the population) have diabetes. Type 2 diabetes (T2D) is the most common form of DM and accounts for 90–95% of all diabetic cases. In T2D, pancreatic islet β-cell function is impaired by a combination of genetic and environmental factors, resulting in insufficient insulin secretion and gradual loss of β-cell mass over time [1,2,3]. When insulin secretion no longer meets metabolic demand, blood glucose continues to rise to a level where frank diabetes is diagnosed. In type 1 diabetes (T1D), islet beta cells are killed by autoimmune attack, so T1D patients have to rely on daily insulin injection.

Given the central importance of the islet β-cell in insulin secretion and euglycemia maintenance, there has been strong interest and intense effort in identifying signaling pathways and therapeutic targets to maintain β-cell fitness, prevent its loss, restore its function, and/or to regenerate β-cells for treating DM [4,5,6]. While the specific triggering events causing both forms of diabetes remain largely undetermined, studies over the past few decades have highlighted the role played by ceramides in β-cell apoptosis, especially under glucolipotoxic and inflammatory conditions. This review focuses on sphingolipids, ceramides in particular, and adiponectin receptor signaling; summarizes recent advancements in our understanding of their roles in islet β-cells, and discusses the therapeutic potential of targeting these signaling events to prevent β-cell loss. Two general pharmacological approaches for regulating ceramide metabolism are explored. These are inhibiting ceramide synthesis using inhibitors of enzymes involved in ceramide production [7], and promoting ceramide degradation using agonists to activate adiponectin receptors and associated ceramidase activity [8].

## 2. Sphingolipid and Ceramide Metabolism

**Figure 1:**
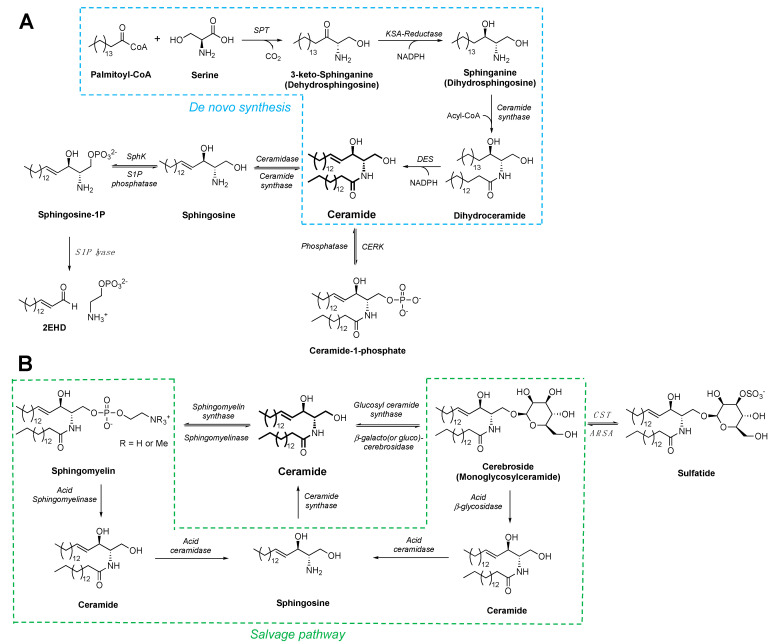
Synthesis and metabolism of sphingolipids and ceramides. (**A**) De novo synthesis of sphingolipids begins with the condensation of serine and palmitate to 3-ketosphinganine, which is reduced to sphinganine. Ceramide synthase catalyzes the N-acylation of sphinganine to dihydroceramide, which forms ceramide after desaturation. SPT: serine palmitoyltransferase; KSA-reductase: 3-ketosphinganine reductase; DES: dihydroceramide desaturase; SphK: sphingosine kinase; S1P phosphatase: sphingosine 1-phosphate phosphatase; 2EHD: (2*E*)-hexadecenal; CERK: ceramide kinase. (**B**) Salvage pathway of ceramide generation. Sphingomyelin and cerebroside are produced from ceramide. They can be trafficked to the acidic compartments, including late endosomes or lysosomes, and hydrolyzed to ceramide and sphingosine, which is then recycled to produce ceramide. Monoglycosylceramides include glucosylceramides (not shown) and galactosylceramides (shown). The latter can be reversibly converted to sulfatide by cerebroside sulfotransferase (CST) or back to cerebroside by arylsulfatase A (ARSA, cerebroside sulfatase).

Sphingolipids are a distinct class of lipids defined by their 18-carbon amino alcohol backbone. Ceramides represent the central hub of the sphingolipid network, and function as key intermediates and precursors for making other sphingolipids (Figure 1). The development of the Lipid Maps Lipidomics Gateway consortium (https://www.lipidmaps.org/, accessed on 1 September 2022), spearheaded by Dr. Edward Dennis and colleagues [9], has provided a valuable resource to categorize numerous classes of lipids, including thousands of distinct sphingolipids [10]. Lipid Maps is a free, comprehensive online resource serving the lipid-research community, providing tutorials, protocols, and standards and databases of lipid compositions and lipid-associated proteins and enzymes to facilitate investigating the roles of diverse lipid molecules in biology and human diseases [11].

In mammalian cells, sphingolipids are less abundant than glycerophospholipids, and constitute roughly 8% of the entire lipid pool in cells [12,13]. In human cells, sphingomyelin is the most abundant form, representing ~85% of all sphingolipids, and typically makes up ~15% of the plasma membrane lipids. Sphingomyelin is preferentially distributed to the outer leaflet of the eukaryotic plasma membrane [14], and plays important roles in maintaining membrane mechanical stability, forming micrometer-scale domains and molecular sorting at the plasma membrane [15]. In addition to functioning as structural components of cellular membranes, sphingolipids, ceramides and sphingosine 1-phospahte (S1P) in particular can exert potent biochemical activities in cells and function as signaling molecules (*vide infra*).

Ceramides stand as the central compounds of sphingolipid synthesis and metabolism. De novo ceramide synthesis starts at the cytoplasmic leaflet of the endoplasmic reticulum membrane [16]. Three enzymes—serine palmitoyltransferase (SPT), 3-ketodihydrosphingosine reductase, and (dihydro)ceramide synthase—act in tandem to synthesize dihydroceramides from cytosolic palmitoyl-CoA and serine (Figure 1A). The reaction catalyzed by SPT represents the rate-limiting step of ceramide de novo synthesis. Dihydroceramides are then converted to ceramides by the dihydroceramide desaturases (DES1 and DES2).

Once produced in the ER, ceramides are transported from the ER to the Golgi apparatus via the vesicular transport or through the ceramide transport protein (CERT). In the Golgi lumen, ceramide is converted to sphingomyelin or glycosylceramide (including glucosylceramide and galactosylceramide, Figure 1B). These sphingolipids are localized to the Golgi luminal leaflet, leading to their preferential distribution to the outer leaflet of the plasma membrane once the vesicles are trafficked to the cell surface. Physiological levels of sphingolipids may serve diverse functions. Ceramides, for instance, have been found in ceramide-rich microdomains along the cell membrane. These ceramide-enriched membrane platforms are thought to be involved in receptor clustering and organizing cellular signaling [17]. Galactosylceramide can also be converted by the cerebroside sulfotransferase to sulfatide, which is found in relatively high abundance in the plasma membrane of neurons and islet β-cells, and in the membrane of insulin granules [18]. Sulfatide has been reported to act as an insulin chaperone and to regulate insulin secretion by affecting the gating of ATP-sensitive potassium channels (K_ATP_ channel) [19,20]. Besides de novo synthesis, ceramides can be generated through the salvage pathway directly from sphingosine that is produced from sphingolipid hydrolysis in the lysosome (Figure 1B). Ceramide synthase, the same enzyme for synthesizing dihydroceramide in the ER, is responsible for the production of ceramide from sphingosine in the salvage pathway. Both de novo and salvage pathways contribute to the ceramide production in islet β-cells [21].

Mammalian ceramide synthase (CerS, also known as Lass) exists in six isoforms, CerS1 to CerS6. These enzymes exhibit preferences for specific fatty acid chain lengths during the acylation reaction (Table 1). CerS1 attaches C18 fatty acyl-CoA to the sphingoid base, and overexpression of CerS1 results in a selective increase in C18 ceramide in cells [22]. CerS2 attaches long fatty acyl-CoAs, such as C22–C24, while CerS3 attaches very long C26–C34 acyl-CoA. CerS4 prefers C18–C20 fatty acyl-CoA, and CerS5 and CerS6 are more selective for C14–C16 acyl-CoA [23]. Since ceramides containing different fatty acids may have distinct impacts on cell physiology, modulating the activity of a specific CerS enzyme is expected to affect the acyl-chain composition of cellular ceramides, hence their associated cell signaling events. Even though both CerS5 and CerS6 contribute to C16 ceramide synthesis, only CerS6-derived C16 sphingolipids bind the mitochondrial fission factor and promote mitochondrial fragmentation, suggesting that CerS5 and CerS6 regulate distinct ceramide pools with nonoverlapping functions [24]. CerS2, CerS4, CerS5, and CerS6 are expressed in islet β-cells.

Through the action of ceramide kinase (CERK), ceramide is phosphorylated to ceramide-1-phosphate, a molecule that has been proposed to play a role in cell survival and inflammation [25]. Its role in inflammation is in part mediated through the activation of cytosolic phospholipase A2 [26], a key enzyme responsible for the production of arachidonic acid and the downstream inflammatory mediator prostaglandins [27]. Besides phosphorylation, ceramides can be hydrolyzed by ceramidases to sphingosine, which can be converted to sphingosine 1 phosphate (S1P) by sphingosine kinases. Compared to other phosphorylated signaling lipids such as phosphoinositides that act as intracellular messengers, S1P is unique in that it is exported outside of cells by S1P transporters, including ATP-binding cassette transporters and SPNS2. Once in the extracellular space, S1P acts on its receptors, S1PRs, to function in an autocrine or paracrine fashion [28]. S1PRs (S1PR1 to S1PR5) are G-protein-coupled receptors (GPCRs) with tissue-specific expression [29]. Depending on the cell type and tissue, S1P may play diverse signaling roles through S1PRs and their downstream proteins, including protein kinase Akt and Rho GTPases, which may support cell survival or regulate the cytoskeleton, respectively [30]. S1P-S1PR signaling plays an important role in the immune system, and targeting S1P signaling has led to several drugs for treating immune-mediated diseases [31]. In addition to its action on cell-surface S1PRs, S1P may function intracellularly to directly regulate cytosolic targets, including histone deacetylases (HDACs) [28]. S1P can be degraded by several enzymes: either irreversibly by S1P lyase into 2E-hexadecenal (2EHD) and phosphoethanolamine (PE), which are used for glycerolipid synthesis, or reversibly dephosphorylated back to sphingosine by S1P-specific phosphatase or nonspecific lipid phosphate phosphatases (LPPs) (Figure 1A). LPPs may act extracellularly to attenuate S1P-stimulated S1PR signaling through their ectoenzyme activity. In addition, LPPs may hydrolyze S1P intracellularly to regulate the intracellular level of S1P [32].

Levels of cellular and circulating ceramides are regulated by both physiological and pathophysiological changes. Inflammation and inflammatory cytokines including tumor necrosis factor a (TNF-a) and interleukins increase ceramide synthesis, and serum circulating levels of IL-6 are strongly correlated with plasma ceramide concentrations [33]. Production of ceramides can be further enhanced by a synergistic action of fatty acids and lipopolysaccharide (LPS) [34,35]. Mechanistically, LPS activates Toll-like receptor-4 (TLR4), which in turn upregulates the expression of enzymes involved in the ceramide synthesis [36].

## 3. Ceramide Hydrolysis by Ceramidases and Adiponectin Receptors

Ceramides are degraded by ceramidases, a group of five enzymes characterized by their pH optima [37], including one acid ceramidase (ASAH1), one neutral ceramidase (ASAH2), and three alkaline ceramidases (ACER1, ACER2, and ACER3). ASAH1 is ubiquitously expressed in mammalian cells and is localized in the lysosome [38]. Secreted or nuclear-localized ASAH1 has also been reported [39,40]. Other ceramidases display tissue or cell specific expression and are localized to different cellular compartments. This family of enzymes play diverse roles in regulating various biological processes through their effects on metabolizing sphingolipids [37].

Besides these canonical ceramidases, adiponectin receptors (ADIPORs), including ADIPOR1 and ADIPOR2, have been shown to possess ceramidase activity [41,42]. Adiponectin is a 30 KDa protein secreted by adipocytes. This cytokine contains 244 amino acids and exhibits pleiotropic metabolic effects, including insulin sensitization, anti-inflammation, and antiapoptosis in multiple tissue types, including muscle, liver, fat and pancreas, among others [43]. Consistent with its metabolic benefits, the reduced level of adiponectin in the circulation is correlated with a number of human malignancies, including diabetes, inflammation, obesity, fibrosis, and cardiovascular disease. Its receptors, ADIPOR1 and ADIPOR2 [44], belong to the family of progestin and adipoQ receptors (PAQR), a group of cell surface receptors related to but distinct from the G protein-coupled receptors (GPCR). Both GPCR and PAQR have seven transmembrane proteins, but PAQRs have their N-terminus facing the cytosol and GPCRs have the C-terminus facing the cytosol [45]. Adiponectin, when overexpressed by two- to threefold, is able to maintain β-cell mass and glucose homeostasis in a genetic *obese* mouse model (*ob*/*ob* mice) [46]. Adiponectin is therefore regarded as a promising antidiabetic adipokine.

**Figure 2:**
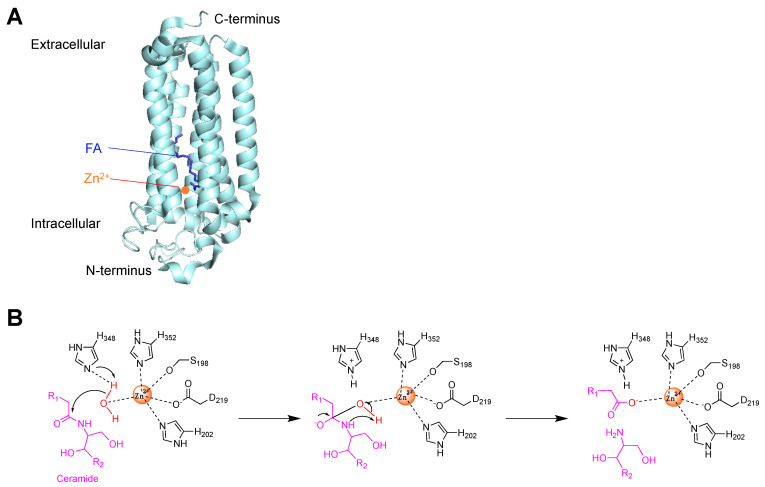
ADIPORs are Zn^2+^-dependent ceramidases. (**A**) X-ray crystal structure of ADIPOR2 in complex with a fatty acid (FA) and Zn^2+^. Based on PDB structure 5LX9. (**B**) Proposed catalytic mechanism of ADIPOR2 ceramidase activity. Zn^2+^ activates a water molecule to attack the amide carbonyl group to form a tetrahedral intermediate. Based on [34].

High-resolution X-ray crystal structures of ADIPORs provide mechanistic insights on the ceramidase activity of these receptors [42]. ADIPORs contain three conserved histidines and one aspartate that are found in the CREST family of metal-dependent transmembrane hydrolases [47]. Two of these histidines and the aspartate are involved in the binding of a zinc ion (Zn^2+^) in their active sites, which contain a hydrophobic binding pocket situated within the transmembrane region (Figure 2). The third histidine and Zn^2+^ activate a water molecule to attack the amide group of ceramide to form a tetrahedral intermediate, which then breaks down to release a fatty acid and sphingosine (Figure 2B).

Similarly to ADIPORs, two ceramidases, ASAH2 and ACER3, are also known to contain Zn^2+^-dependent catalytic sites. Human neutral ceramidase (ASAH2) is a single transmembrane protein, yet it contains a narrow, hydrophobic pocket with a Zn^2+^ ion [48]. Two histidines and one glutamic acid form the Zn^2+^-coordination pocket. During catalysis, the Zn^2+^ ion activates a water molecule for the nucleophilic attack of the amide bond. Human alkaline ceramidase 3 (ACER3), despite a very low sequence identity with ADIPORs (14% with ADIPOR1 and 10% for ADIPOR2), has a protein fold similar to ADIPORs with seven transmembrane helices harboring a Zn^2+^ binding site that contains three histidines [49]. Again, the Zn^2+^ ion coordinates with a water molecule to activate the water for amide hydrolysis. The X-ray structure and computational docking analysis suggest that the hydrophobic substrate binding pocket of ACER3 can only accommodate ceramides, whereas sphingolipids containing a polar head group larger than the hydroxy of ceramides, such as sphingomyelin, glucosylceramide or ceramide-1-phosphate, cannot be accommodated in the pocket. Together, these structural studies highlight a conserved mechanism for the Zn^2+^-dependent ceramide hydrolysis by these lipid amidases.

## 4. Ceramide in Cell Stress and Apoptosis of Islet β-Cells

**Figure 3:**
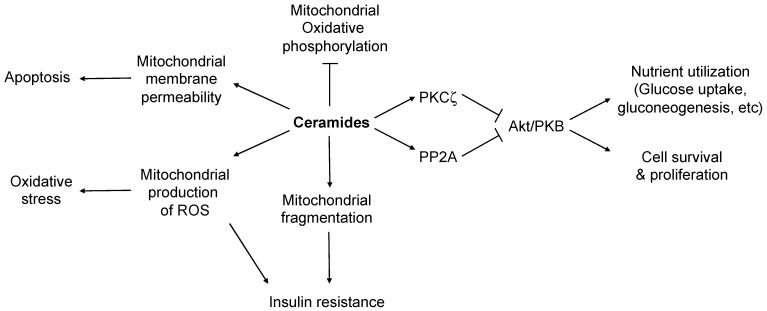
Modes of action of ceramides causing insulin resistance and apoptosis. Ceramides may cause insulin resistance through direct inhibition of the insulin signaling pathway. Elevated C16:0-ceramide is believed to be particularly harmful in causing mitochondrial dysfunction, while elevated C18:0-ceramide has been shown to be a major player in insulin resistance in skeletal muscle. See the main text for details.

Elevated levels of ceramides, especially ceramides containing a C16:0 acyl chain [50], are harmful to cells, and can elicit metabolic dysfunctions, including insulin resistance, dyslipidemia, and even cell death. Concentrations of ceramides in the blood or in fat and liver tissue are strongly correlated with insulin resistance, the risk of developing T2D, and liver diseases, including hepatic steatosis and nonalcoholic steatohepatitis (NASH) [51,52,53]. Mechanistically, ceramides inhibit insulin action by preventing Akt kinase activation. Akt is a serine/threonine kinase that enhances glucose usage and promotes cell survival. Through acting on protein phosphatase 2A (PP2A) [54,55,56], and/or on protein kinase Cζ (PKCζ) [57], ceramides modulate the phosphorylation state of Akt to regulate its activity or subcellular localization (Figure 3). In skeletal muscle, C18:0 ceramide has been shown to be strongly correlated with insulin resistance [58,59]. In addition to its role in inhibition of insulin signaling, ceramides, C16:0 ceramide in particular [60,61,62], reduce the electron transport chain activity of mitochondria [50,61,62,63] and increase mitochondrial outer membrane permeability, causing cytochrome *c* release and apoptosis [64,65,66,67,68]. C16 ceramide also induces the formation of reactive oxygen species (ROS) by modulating the activity of mitochondrial complex IV, resulting in chronic oxidative stress [62]. Moreover, C16:0 ceramide has been shown to bind to mitochondrial fission factor to promote mitochondrial fragmentation that contributes to insulin resistance [24].

Obesity is an established risk factor of T2D. Obesity and elevated fatty acids can cause insulin resistance through a number of factors, including the increased production of proinflammatory cytokines such as TNFα and interleukins, the reduced level of adiponectin, and the increased accumulation of ceramides in tissue. In addition, obesity and elevated fatty acids can directly impact islet β-cells by promoting β-cell apoptosis, a process where ceramides were shown to play a key role [69,70]. In the Zucker diabetic fatty (ZDF) rat, a rodent model of obesity and T2D, ceramides were significantly increased in prediabetic and diabetic islets. In vitro, C2 ceramide induced apoptosis, whereas inhibitors of ceramide synthesis blocked fatty acid-induced apoptosis in cultured ZDF islets [69]. In addition to their effect on apoptosis, ceramides were also reported to negatively impact β-cell function by compromising insulin secretion and reducing insulin gene expression [71,72]. Ceramide accumulation is a key driver of β-cell ER stress, and ceramide accumulation has been suggested to alter ER membrane properties, reduce ER protein export and perturb ER calcium handling or signaling [73]. Consistently with the toxic effect of ceramide accumulation, genetic ablation of Sphk1 disrupted the homeostatic flux between ceramide and S1P (Figure 1), and predisposed diet-induced obese mice to diabetes by promoting pancreatic β-cell death, while S1P bolstered resistance to palmitate-induced cell death [74]. Moreover, expression of dominant-negative SphK1 promoted palmitate-induced β-cell death. These data support a beneficial role of SphK1 in protection from lipotoxicity-induced pancreatic dysfunction.

A number of studies have consistently shown an antiapoptotic effect of adiponectin in islet β-cells in vitro, under settings of elevated fatty acids or serum starvation. These results are in line with the ceramide lowering activity of ADIPORs, and appear to be independent of AMPK activation [8,41,75]. Adiponectin has also been reported to stimulate insulin secretion in vitro and in vivo at basal glucose concentration [75,76]. In the islets of Langerhans, both ADIPOR1 and of ADIPOR2 are expressed, with ADIPOR1 being the major isoform expressed in both mouse and human β cells [75,77,78]. In a mouse insulinoma cell line (MIN6 cells) and in isolated mouse islets, serum starvation caused β-cell death. Adiponectin improved cell viability and reduced apoptosis [75]. The mechanism of protection was independent of AMPK activation, confirming that AMPK signaling is not universally required in the action of adiponectin or ADIPORs. In a rat β-cell line (INS1 cells), adiponectin exerted potent protection against both palmitic acid-induced and cytokine-induced (IL-1β and IFN-γ) apoptosis [79]. In addition to its antiapoptotic activity, adiponectin also increases insulin content and cell proliferation of MIN6 cells, and increases glucose-stimulated insulin secretin (GSIS) in isolated rat islets [80,81,82].

Besides these in vitro studies, a number of in vivo studies have consistently shown an antiapoptotic effect of adiponectin in islet β-cells under conditions mimicking either type 1 or type 2 diabetes. Scherer’s group identified a novel mechanism of β-cell protection by adiponectin that involves adiponectin-mediated activation of the ceramidase activity of ADIPORs, as mentioned earlier. This reduces cytotoxic ceramides to mitigate lipotoxicity [41]. In vivo, injected adiponectin bound to islet β-cells avidly and accumulated in mouse islets. To examine the protective effect of adiponectin on β-cells, they resorted to an inducible β-cell apoptosis transgenic mouse termed PANIC-ATTAC mouse. Compared to the PANIC-ATTAC mice expressing adiponectin, adiponectin-null PANIC-ATTAC showed a drastic reduction in islet β-cell mass, highlighting the role of adiponectin in maintaining β-cell mass in vivo in this mouse model of β-cell loss mimicking diabetes [41]. Mechanistically, adiponectin improves β-cell maintenance and promotes β-cell regeneration and proliferation by mitigating lipotoxicity both systemically and locally in pancreatic islets [83,84]. Given the clinical importance of developing new approaches for β-cell regeneration, it would be important to devise strategies to selectively activate ADIPORs in islet β-cells to enhance the β-cell autonomic effect of adiponectin and ADIPOR signaling.

In addition to its well-documented involvement in β-cell degeneration in lipotoxicity and T2D, ceramides are also implicated in the β-cell demise of T1D. T1D is caused by the autoimmune destruction of pancreatic β cells due to the enhanced production of interleukins and other proinflammatory cytokines. Proinflammatory cytokines, particularly interleukin-1β (IL-1β), interferon γ (IFNγ), and TNFα, have been implicated in the killing of β-cells [85]. The mechanisms underlying β-cell death due to the exposure of these cytokines are complex. In addition, IL-1β, IFNγ and TNFα are known to cause β-cell dysfunction and reduce insulin synthesis. Addition of exogenous ceramide or stimulation of endogenous ceramide production mimic some of the cytotoxic and insulinopenic effects of proinflammatory cytokines on β-cells [86], and some (but not all) studies have reported the increased ceramide production in β-cells upon exposure to inflammatory cytokines [87,88,89]. In addition to increasing ceramide production, downregulation of ceramide degradation may also be involved in the cytotoxicity of proinflammatory cytokines. Phosphorylation of sphingosine into S1P and subsequent degradation of S1P by S1P lyase are important transformations to keep the level of sphingosine and ceramide in check. In INS1 β-cells, proinflammatory cytokines increase ceramide and sphingosine production, but reduce S1P lyase expression, shifting the equilibrium towards ceramide and sphingosine accumulation [90]. Overexpression of S1P lyase protects β-cells from cytokine toxicity and reduces ER stress, implicating a therapeutic strategy for T1D by reducing ceramide and sphingosine levels through S1P lyase activation [90].

## 5. Targeting Ceramide Metabolism by Inhibiting Ceramide Synthesis to Rescue β-Cells

**Figure 4:**
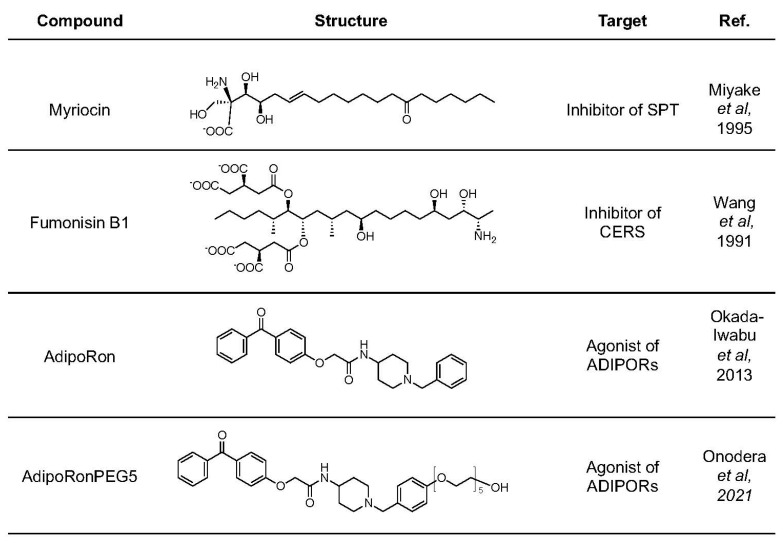
Pharmacological agents targeting ceramide metabolism and adiponectin receptors. Corresponding references for these compounds are [91], [93], [103], and [8], respectively (from top to bottom).

Several small molecules have proven to be useful pharmacological reagents for investigating ceramide metabolism and probing ceramide functions in vitro and in vivo (Figure 4). Myriocin (ISP-1) is a sphingosine homologue. It potently inhibits serine palmitoyltransferase (SPT) [91]. Since SPT initiates the de novo synthesis of ceramides, myriocin is especially useful for dissecting the de novo pathway from the salvage pathway of ceramide synthesis [21]. In isolated human islets exposed to free fatty acids, myriocin partially prevented β-cell apoptosis, confirming the involvement of ceramide production in β-cell death [92]. Fumonisins are natural products from *Fusarium* fungus. They are structural analogues of sphingosine and potent inhibitors of ceramide synthases (CerSs) [93]. In prediabetic and diabetic Zucker diabetic fatty (ZDF) rat islets, apoptosis was increased by up to sevenfold compared to the nondiabetic controls. Treatment with fumonisin B1 blocks free fatty acids induced apoptosis in ZDF islets in vitro [69]. In MIN6 cells or isolated islets exposed to palmitate, fumonisin B1 blocked the deleterious effects of palmitic acid on β-cell viability [21,94], confirming the involvement of CerSs in producing toxic ceramides during palmitate treatment and supporting a therapeutic strategy of preventing lipotoxicity and β-cell death by inhibiting CerSs.

Lipotoxicity and ceramide can also affect hypothalamic neurons to induce insulin resistance and dysregulation of glucose homeostasis [95]. In vitro, palmitate impairs insulin signaling and increased ceramide levels in hypothalamic neuronal GT1-7 cells, while myriocin treatment restores insulin signaling in palmitate-treated GT1-7 cells. In vivo, obese Zucker rats were intracerebroventricularly infused with myriocin to inhibit de novo ceramide synthesis. This treatment attenuated the increase in levels of hypothalamic ceramides and improved hypothalamic insulin sensitivity. Moreover, central myriocin treatment partially restored glucose tolerance in obese Zucker rats. This effect appeared to be related to the restoration of glucose-stimulated insulin secretion and an increase in β-cell mass, suggesting that hypothalamic ceramides can regulate insulin secretion and even β-cell mass by inhibiting parasympathetic activity [96].

Besides SPT and ceramide synthases, other targets for reducing ceramide production include dihydroceramide desaturase (DES) and sphingomyelinase (Figure 1). Potent inhibitors have been developed against these two classes of enzymes as candidate therapeutic agents against a variety of diseases including cancer, Alzheimer’s disease, and diabetes [7]. In addition, β-glycosidases, including glucocerebrosidase and galactocerebrosidase, generate ceramides from the corresponding cerebrosidases (Figure 1). These glycosidases can be potently inhibited by the class of chemicals termed iminosugars [97,98]. However, unlike myriocin or fumonisin B1, the effects of these different classes of inhibitors of ceramide production on islet β-cells have not been extensively investigated. Future applications of these compounds in β-cells may shine light on the roles of DES, sphingomyelinase, and glucocerebrosidase on β-cell survival or function in the context of ceramide regulation and metabolism.

## 6. Activating ADIPORs to Degrade Ceramides and Promote β-Cell Survival and Function

The pleiotropic benefits of adiponectin in diverse human organs and tissue supports its therapeutic potential in correcting several metabolic diseases. However, adiponectin forms higher-order structures through multimerization. These higher-order structures include trimers, hexamers, and multimers (12–18 units of adiponectin), with the high-molecular-weight multimer of adiponectin being the most biologically active form [99,100,101]. From a therapeutic standpoint, the formation of higher-order complexes complicates the formulation of adiponectin protein as a drug molecule. This limitation of adiponectin protein has motivated the development of adiponectin agonists based on peptides [102] or small molecules. Through a high-throughput screening of a small-molecule library, Kadowaki’s group identified AdipoRon as the first small-molecule agonist of ADIPOR1 and ADIPOR2 (Figure 4) [103]. In vitro, AdipoRon activated the AMPK pathway. In vivo, it ameliorated insulin resistance and improved glucose tolerance in mice fed a high-fat diet or in a genetic *diabetes* mouse model (*db*/*db* mice). In addition, AdipoRon reduced triglyceride content and oxidative stress in the liver by increasing the expression level of antioxidative stress genes, such as manganese superoxide dismutase [103]. Despite its beneficial effects in mouse models, AdipoRon’s therapeutic potential is hampered by its low aqueous solubility. Even in a neutral phosphate buffer containing >30% DMSO, AdipoRon is only soluble to 0.25 mg/mL [104].

**Figure 5:**
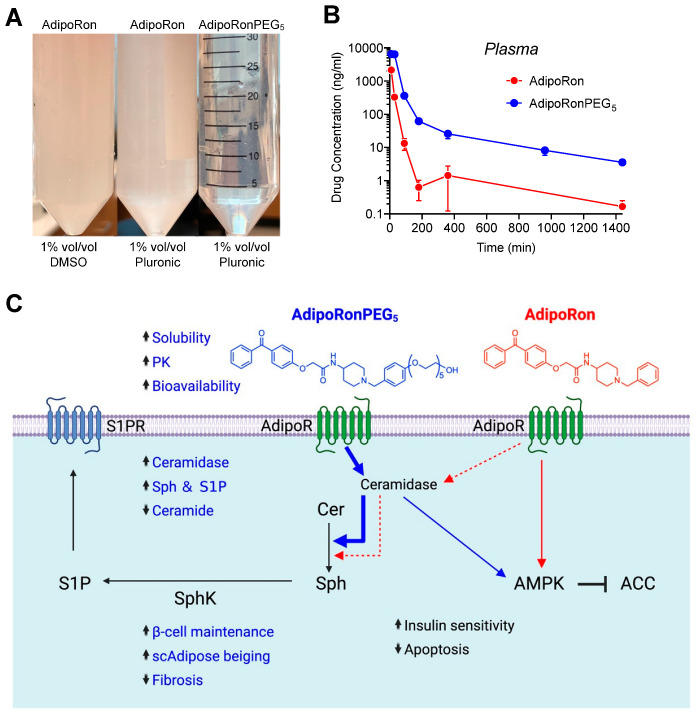
Pharmacological improvements of AdipoRonPEG_5_ enhance its action through ADIPOR to exert pleiotropic benefits on metabolism, cell function, and survival. (**A**) Aqueous solubility of AdipoRonPEG5 is drastically enhanced. (**B**) Pharmacokinetic profiles of AdipoRon and AdipoRonPEG_5_ upon IP dosing at 20 mg/kg in C57BL/6J mice. (**C**) Comparison of cellular actions of AdipoRonPEG_5_ and AdipoRon. Adapted from [8] with permission from Elsevier.

To improve the aqueous solubility and biodistribution of AdipoRon, we developed AdipoRonPEG_5_ as a hydrophilic analogue of AdipoRon (Figure 4) [8]. AdipoRonPEG_5_ contains a pentaethylene glycol moiety that boosts the water solubility of AdipoRonPEG_5_ by about 100 times over AdipoRon (910 ± 36 μg/mL for AdipoRonPEG_5_ vs. 8.99 ± 0.15 μg/mL for AdipoRon, Figure 5A). This led to an enhanced bioavailability in the circulation after intraperitoneal injection (IP), achieving a threefold increase in the maximal plasma concentration of AdipoRonPEG_5_ compared to AdipoRon (Figure 5B). The difference in the bioavailability of these drugs in the mouse liver was even more dramatic, with AdipoRonPEG_5_ showing eightfold bioavailability in the liver [8]. In INS-1 β-cells treated with palmitate, both AdipoRonPEG_5_ and AdipoRon exerted a potent antiapoptosis effect, yet only AdipoRonPEG_5_ but not AdipoRon markedly lowered overall dihydroceramide and ceramides levels, demonstrating the superior effect of AdipoRonPEG_5_ on ceramide reduction in INS1 β-cells [8]. Gene expression analysis of INS-1 cells showed that AdipoRonPEG_5_ significantly increased the expression of genes that are involved in maintaining the functional state of pancreatic β-cell. These results demonstrate that AdipoRonPEG_5_, compared to AdipoRon, enhanced protective actions against palmitate mediated β-cell cytotoxicity to improve the functional integrity of β-cells under lipotoxic conditions.

In vivo, AdipoRonPEG_5_ displayed a similar effect in reducing ceramide level in both the mouse liver and pancreas under a high-fat diet (HFD) [8]. In parallel with its effect on ceramide reduction, AdipoRonPEG_5_ treatment also altered S1P levels in the mouse pancreas, with a trend of elevating S1P. These results are in line with earlier reports that adiponectin lowered ceramide levels and elevated S1P concentrations in different tissues, further supporting the role of adiponectin and ADIPOR agonists in initiating a receptor-mediated activation of ceramidase activity [41,105].

## 7. Outlook

Over the last few decades, significant efforts and progress have been made in the sphingolipid field to identify mechanisms that can lead to β-cell demise, and to explore pathways for counteracting these events to rescue β-cells. Ceramides are now recognized as key lipid molecules involved in controlling ER stress and β-cell apoptosis in the presence of lipotoxicity and/or proinflammatory cytokines. Under these conditions, adiponectin exhibits a robust antiapoptotic, proliferative activity for protecting β-cells. The success of developing AdipoRon and more recently AdipoRonPEG5 as small-molecule agonists of ADIPORs has paved the way to exploring the targeted delivery of these compounds to trigger enhanced activation of ADIPORs in pancreatic islets to protect β-cells from insults, such as seen in the context of both forms of diabetes. AdipoRon was discovered from a cell-based screening of AMPK activation. Given the primary mode of action of lipid hydrolase of ADIPORs, additional chemical screenings focusing on ceramide hydrolysis may identified new chemical scaffolds with more potent activity in promoting the Zn^2+^-dependent hydrolyase activity of the receptor. Besides ceramides, can other lipids serve as the substrates of ADIPORs, and if so, how does the hydrolysis of those lipid substrates mediate the signaling and metabolic benefits of adiponectin? Answers to such questions are expected to provide fresh insights into the biology of ADIPORs and to stimulate developing new pharmacological agents for treating diabetes.

## Figures and Tables

**Table 1:** Ceramide synthase isoforms and substrate specificity.

Ceramide Synthase (CerS)	Fatty Acids Preference
CerS1	C18
CerS2	C22–24
CerS3	≥C26
CerS4	C18–C20
CerS5	C14–16
CerS6	C14–16
